# A focus on molecular representation learning for the prediction of chemical properties

**DOI:** 10.1039/d4sc90043j

**Published:** 2024-03-25

**Authors:** Yonatan Harnik, Anat Milo

**Affiliations:** a Department of Chemistry, Ben-Gurion University of the Negev Beer Sheva 84105 Israel anatmilo@bgu.ac.il

## Abstract

Molecular representation learning (MRL) is a specialized field in which deep-learning models condense essential molecular information into a vectorized form. Whereas recent research has predominantly emphasized drug discovery and bioactivity applications, MRL holds significant potential for diverse chemical properties beyond these contexts. The recently published study by King-Smith introduces a novel application of molecular representation training and compellingly demonstrates its value in predicting molecular properties (E. King-Smith, *Chem. Sci.*, 2024, https://doi.org/10.1039/D3SC04928K). In this focus article, we will briefly delve into MRL in chemistry and the significance of King-Smith's work within the dynamic landscape of this evolving field.

## Abstract 2

Molecular representation learning (MRL) holds significant potential for predicting diverse chemical properties. In this focus article, we will provide context for applications of MRL in chemistry and the significance of King-Smith's recently published work within this evolving field.

## Graphical abstract

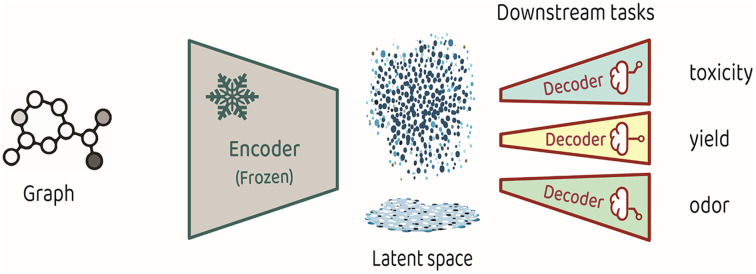

The capacity of a model to extract information from existing data for the prediction of unseen data is at the basis of machine learning. Accordingly, the accuracy of a model depends on its ability to identify the details that best capture a predicted property within the data it is trained on. Numerous manual and automated techniques have been developed for extracting key features from any provided data, a process which is known as featurization. Traditional approaches for featurization in chemistry primarily concentrate on representing reactions and molecules through explicit chemical properties.^1^ Such features are known as molecular descriptors and can be derived from direct experimental measurements or theoretical computational methods such as DFT and quantum mechanics.^2^

In contrast to these methods, molecular representation learning (MRL) introduces an alternative approach to capturing molecular information. Representation learning is a field in machine learning that deals with extracting effective features from raw data using deep-learning models.^3,4^ The objective of such models is to encode the data into a vectorized space designed to create a concise and well-organized map of the input data. Representation learning has recently seen several implementations in chemistry for the featurization of molecules and reactions.^5^ Due to its underlying organized and hierarchical feature space, representation learning can potentially improve a model's ability to predict various chemical properties. Moreover, by allowing a learning algorithm to discover the fundamental factors that define a certain dataset, it can potentially provide non-intuitive molecular descriptions and insights compared to traditional feature extraction approaches.^3^

Training an encoder is at the core of representation learning models. An encoder compresses input data into a latent space, which serves as a vectorized representation of the input, capturing its essential features.^3,4^ The key characteristic of representation learning is that the encoder is created by a training process known as pretraining, which is performed on a task that is suited for identifying the fundamental structure of the input. Some pretraining tasks necessitate a decoder that takes the compressed representation from the latent space and uses it to make predictions (see Fig. 1A for a conceptual architecture of a pretraining model). Alternatively, some pretraining approaches focus only on optimizing the organization of the latent space itself, eliminating the need for a decoder.

**Fig. 1:**
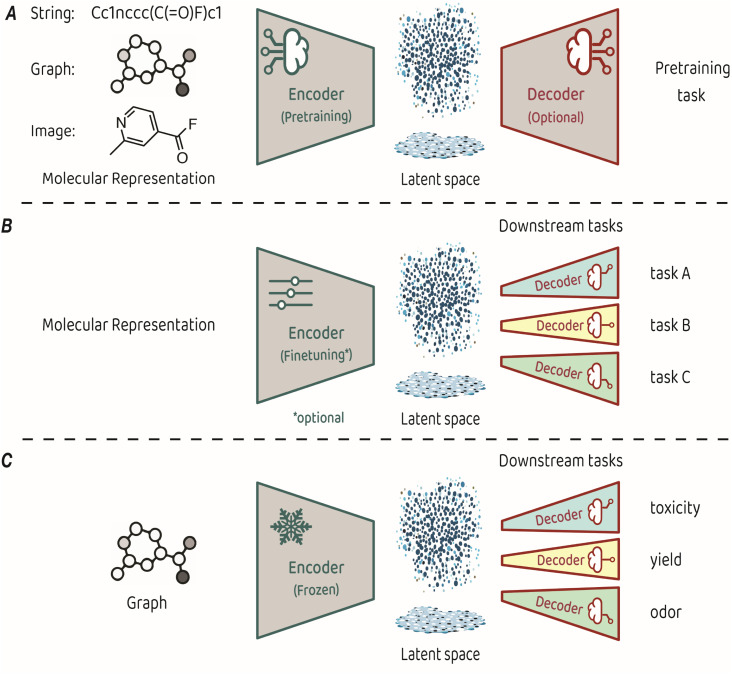
(A) Typical architecture for molecular representation pretraining. The encoder depends on the input representation and a decoder is sometimes required as part of the pretraining task. (B) Typical architecture for transfer learning. The molecular representation is the same as the one used for pretraining, the encoder can be either finetuned or kept frozen during training, and a dedicated decoder is trained for each downstream task. (C) The transfer learning architecture used in King-Smith's work.^37^

In many cases, the pretraining task is either supervised or self-supervised. In supervised learning, the model is trained on pre-labeled data, where the input is paired with its output labels and the model is explicitly guided to create a map that connects inputs with their corresponding outputs accurately. Unsupervised learning involves training the model on data that is not explicitly labeled, leaving the model to discover patterns and structures in the data without guidance. Self-supervised learning is a subset of unsupervised learning where labels are generated during the training process from the input data. For example, if a model is trained to predict missing parts of corrupt inputs, self-supervised learning can be applied to generate labels by omitting parts of flawless inputs. Achieving successful pretraining usually demands a substantial amount of data to ensure generalizability; thus, self-supervised approaches are commonly employed, as they can accept large datasets as inputs without manual labeling.^4,6^

The design of a pretraining encoder depends on the format of its inputs; for example, molecules or reactions can be represented as graphs, strings, and images.^7,8^ Graphs are mathematical objects comprised of a set of nodes connected by a set of edges. Molecules can be straightforwardly represented as graphs, considering atoms as nodes and bonds as edges.^7,8^ Graph representations are a widely used format for MRL, with numerous studies showcasing the efficacy of graph neural networks (GNNs)^6,9–15^ or graph transformers^16^ for pretraining. String representations, such as SMILES^17^ or SELFIES,^18^ which capture the atoms, bonds, charges, and stereochemistry of a molecule in textual format, have also been explored by MRL encoders. These string-based encoders have been trained by employing recurrent neural networks (RNNs)^19,20^ or transformers.^21–24^ Finally, convolutional neural networks (CNNs) have recently been proposed to encode molecular images as inputs.^25,26^

The representations learned in pretraining are leveraged for making predictions on diverse related tasks, often referred to as downstream tasks. This process, also known as transfer learning, requires attaching a new output decoder on top of the latent space, to make predictions for the downstream task at hand (Fig. 1B).^27^ Typically, downstream decoders are simple and compact, such as logistic regression modules or basic neural network architectures (also known as multi-layer perceptrons). Throughout the downstream training phase, the encoder can either remain frozen or undergo careful finetuning toward the task at hand. The applicability of a pretrained model can span a diverse range of downstream tasks, provided that the features acquired during pretraining relate to the predicted property. For example, pretraining on the prediction of basic atom-level properties, such as formal charge and hybridization, or basic bond-level properties, such as conjugation or stereochemistry, have been used for downstream predicted properties, such as toxicity, protein binding affinity and water solubility.^9^

The recent work by King-Smith proposes a machine-learning framework that leverages graph-based MRL to make accurate predictions in chemistry-related tasks with limited data.^37^ The approach involves pretraining a graph neural network model on a dataset of organic crystal structures from the Cambridge Crystallographic Data Centre (CCDC).^28^ In the pretraining phase, a supervised-learning approach was employed. The model learned to predict the angles and bond lengths of a given molecule, where the ground truths were extracted from the crystallographic data. The input molecules were represented as molecular graphs, which then underwent encoding through a message-passing neural network (MPNN) to reach the latent space, from which the output (angles and bond lengths) was predicted by regression. Pretraining was performed on a dataset of approximately 1 million examples. In the transfer learning phase, the pretrained encoder was applied to smaller, task-specific datasets of a few thousands of samples. These task-related compact models (multi-layer perceptrons with two layers) are trained to predict toxicity, yield, and odor. The training process applied the molecular features acquired by the encoder in the pretraining phase without finetuning, while optimizing the task-specific decoders for precise predictions in the targeted domains (Fig. 1C).

An acute toxicity dataset from Therapeutics Data Commons (TDC)^31^ was selected as a benchmark task evaluated on the regression prediction of LD_50_ values. To guarantee a varied structural representation across the training, validation, and test sets, a scaffold splitting protocol was followed.^29^ This protocol involves extracting the Murcko scaffold for each molecule,^30^ which is the core structure obtained by a rule-based elimination of substituents. Subsequently, molecules sharing identical scaffolds were categorized into the same subset. The most frequently occurring scaffolds were incorporated into the training set, ensuring exposure to common structural motifs during training, while the least common scaffolds were assigned to the test set, facilitating robust evaluation on less familiar structures. In this downstream task, King-Smith's MRL framework outperformed baseline models including random forest, the Gaussian process, and AdaBoost, as well as the state-of-the-art Oloren ChemEngine model.^32^ To showcase the model's efficacy in handling out-of-domain data, a supplementary test set of twelve non-therapeutic molecules was curated. Once again, the model exhibited enhanced performance compared to the baseline models and Oloren ChemEngine, highlighting its robustness across different molecular domains.

For yield prediction, a Suzuki reaction dataset from the US patent literature (USPTO)^33^ and a Buchwald–Hartwig reaction dataset from high-throughput experimentation (HTE)^34^ were taken as benchmarks. These datasets, which probed the influence on yield of several reaction components, such as base, catalyst and substrate, required modification of King-Smith's MRL approach, because the encoder was designed for molecules and not for reactions. To address this issue, the molecular structures of several encoded reaction components were concatenated to describe each reaction. As a result, a different dataset splitting methodology was applied to create a reliable test set such that for every reaction at least one molecule had not been previously seen in the training set. The performance of King-Smith's model was compared to the above-mentioned baseline models, as well as to YieldBERT^35^ and GraphRXN.^36^ For the Suzuki reaction, the results obtained by the model were only slightly better than those of YieldBERT. The inability to significantly outperform existing models was attributed to the noise associated with the varied sources from which the experimental dataset was extracted. To showcase the model's ability to handle more consistent data, the model was trained on the Buchwald–Hartwig dataset, for which the model outperformed YieldBERT by a more significant margin.

The third model evaluation task focused on predicting odors, presenting a challenging multi-classification task with 113 unique odor-type labels. Model performance was assessed based on a standard 5-fold cross validation test and an external dataset of 22 out-of-domain molecules. Whereas this external validation set is very small, it is extremely challenging because it consists of 11 pairs of enantiomers, each of which has a different smell profile. King-Smith's model demonstrated significantly superior performance in both test scenarios compared to two classification baseline models, random forest and *k*-nearest neighbors.

King-Smith introduces an efficient methodology for graph neural network based MRL, showcasing state-of-the-art performance in several tested downstream tasks. This work is a significant contribution to the evolving field of MRL in chemistry and with time should be evaluated across a broader spectrum of downstream tasks and diverse benchmarks. An important aspect of this study is that in contrast to most work in the field of MRL in chemistry, which is primarily focused on drug design, it showcases the broad utility of this approach across a more diverse array of downstream tasks. In this vein, narrowing the gap between MRL and fundamental chemistry principles holds promise for advancing predictive modeling in countless chemistry domains.

## Author contributions

YH and AM wrote the manuscript together.

## Conflicts of interest

There are no conflicts to declare.

## Supplementary Material

## References

[cit1] Consonni V. and Todeschini R., Molecular descriptors. Challenges and Advances in Computational Chemistry and Physics, 2010, vol. 8, pp. 29–102

[cit2] Singh S., Sunoj R. B. (2023). Molecular Machine Learning for Chemical Catalysis: Prospects and Challenges. Acc. Chem. Res..

[cit3] Bengio Y., Courville A., Vincent P. (2013). Representation learning: a review and new perspectives. IEEE Trans. Pattern Anal. Mach. Intell..

[cit4] Ericsson L., Gouk H., Loy C. C., Hospedales T. M. (2022). Self-Supervised Representation Learning: Introduction, Advances, and Challenges. IEEE Signal Process. Mag..

[cit5] Li Z., Jiang M., Wang S., Zhang S. (2022). Deep learning methods for molecular representation and property prediction. Drug Discovery Today.

[cit6] Zhou G. , et al., Uni-Mol: A Universal 3D Molecular Representation Learning Framework, 2022, 10.26434/CHEMRXIV-2022-JJM0J

[cit7] Wigh D. S., Goodman J. M., Lapkin A. A. (2022). A review of molecular representation in the age of machine learning. Wiley Interdiscip. Rev.: Comput. Mol. Sci..

[cit8] David L., Thakkar A., Mercado R., Engkvist O. (2020). Molecular representations in AI-driven drug discovery: a review and practical guide. J. Cheminf..

[cit9] Yang K. (2019). *et al.*, Analyzing Learned Molecular Representations for Property Prediction. J. Chem. Inf. Model..

[cit10] Xiong Z. (2020). *et al.*, Pushing the boundaries of molecular representation for drug discovery with the graph attention mechanism. J. Med. Chem..

[cit11] Sun Q. , *et al.*, SUGAR: Subgraph neural network with reinforcement pooling and self-supervised mutual information mechanism, The Web Conference 2021 – Proceedings of the World Wide Web Conference, WWW 2021, 2021, pp. 2081–2091, 10.1145/3442381.3449822

[cit12] Wang H. , *et al.*, Chemical-Reaction-Aware Molecule Representation Learning, ICLR 2022 – 10th International Conference on Learning Representations, 2021

[cit13] Ji Z., Shi R., Lu J., Li F., Yang Y. (2022). ReLMole: Molecular Representation Learning Based on Two-Level Graph Similarities. J. Chem. Inf. Model..

[cit14] Guo Z. , *et al.*, Graph-based Molecular Representation Learning, IJCAI International Joint Conference on Artificial Intelligence 2023-August, 2022, pp. 6638–6646

[cit15] Fang X. (2022). *et al.*, Geometry-enhanced molecular representation learning for property prediction. Nat. Mach. Intell..

[cit16] Rong Y. (2020). *et al.*, Self-Supervised Graph Transformer on Large-Scale Molecular Data. Adv. Neural Inf. Process. Syst..

[cit17] Weininger D. (1988). SMILES, a Chemical Language and Information System: 1: Introduction to Methodology and Encoding Rules. J. Chem. Inf. Comput. Sci..

[cit18] Krenn M., Häse F., Nigam A. K., Friederich P., Aspuru-Guzik A. (2020). Self-referencing embedded strings (SELFIES): A 100% robust molecular string representation. Mach. Learn.: Sci. Technol..

[cit19] Li C., Feng J., Liu S., Yao J. (2022). A Novel Molecular Representation Learning for Molecular Property Prediction with a Multiple SMILES-Based Augmentation. Comput. Intell. Neurosci..

[cit20] Pinheiro G. A., Da Silva J. L. F., Quiles M. G. (2022). SMICLR: Contrastive Learning on Multiple Molecular Representations for Semisupervised and Unsupervised Representation Learning. J. Chem. Inf. Model..

[cit21] Chithrananda S. , Grand G. and Deepchem B. R., ChemBERTa: Large-Scale Self-Supervised Pretraining for Molecular Property Prediction, 2020

[cit22] Wang S. , Guo Y., Wang Y., Sun H. and Huang J., Smiles-Bert: Large scale unsupervised pre-training for molecular property prediction, ACM-BCB 2019 – Proceedings of the 10th ACM International Conference on Bioinformatics, Computational Biology and Health Informatics, 2019, pp. 429–436, 10.1145/3307339.3342186

[cit23] Fabian B. , et al., Molecular Representation Learning with Language Models and Domain-Relevant Auxiliary Tasks, 2020

[cit24] Yüksel A., Ulusoy E., Ünlü A., Doğan T. (2023). SELFormer: molecular representation learning *via* SELFIES language models. Mach. Learn.: Sci. Technol..

[cit25] Iqbal J., Vogt M., Bajorath J. (2021). Learning functional group chemistry from molecular images leads to accurate prediction of activity cliffs. Artif. Intell. Life Sci..

[cit26] Zeng X. (2022). *et al.*, Accurate prediction of molecular properties and drug targets using a self-supervised image representation learning framework. Nat. Mach. Intell..

[cit27] Zhuang F. (2021). *et al.*, A Comprehensive Survey on Transfer Learning. Proc. IEEE.

[cit28] Allen F. H. , et al., The Cambridge Crystallographic Data Centre: Computer-Based Search, Retrieval, Analysis and Display of Information, 1979, vol. 35, urn:issn:0567-7408, pp. 2331–2339

[cit29] Wu Z. (2018). *et al.*, MoleculeNet: a benchmark for molecular machine learning. Chem. Sci..

[cit30] Bemis G. W., Murcko M. A. (1996). The properties of known drugs. 1. Molecular frameworks. J. Med. Chem..

[cit31] Huang K. , et al., Therapeutics Data Commons: Machine Learning Datasets and Tasks for Drug Discovery and Development, 2021

[cit32] Huang D. , et al., A Unified System for Molecular Property Predictions: Oloren ChemEngine and its Applications, 2022, 10.26434/CHEMRXIV-2022-ZZ776

[cit33] Chemical reactions from US Patents (1976-Sep. 2016), https://figshare.com/articles/dataset/Chemical_reactions_from_US_patents_1976-Sep2016_/5104873/1

[cit34] Ahneman D. T., Estrada J. G., Lin S., Dreher S. D., Doyle A. G. (2018). Predicting reaction performance in C–N cross-coupling using machine learning. Science.

[cit35] Schwaller P., Vaucher A. C., Laino T., Reymond J. L. (2021). Prediction of chemical reaction yields using deep learning. Mach. Learn.: Sci. Technol..

[cit36] Li B. (2023). *et al.*, A deep learning framework for accurate reaction prediction and its application on high-throughput experimentation data. J. Cheminf..

[cit37] King-Smith E. (2024). Chem. Sci..

